# Neurophysiological Basis of Electroacupuncture Stimulation in the Treatment of Cardiovascular‐Related Diseases: Vagal Interoceptive Loops

**DOI:** 10.1002/brb3.70076

**Published:** 2024-09-30

**Authors:** Yun Liu, Tiancheng Xu, Zhi Yu, Bin Xu

**Affiliations:** ^1^ Key Laboratory of Acupuncture and Medicine Research of Ministry of Education Nanjing University of Chinese Medicine Nanjing China

**Keywords:** cardiovascular | electroacupuncture stimulation | somatic afferent nerve | vagal interoceptive

## Abstract

**Purpose:**

The vagal sensory nerve (VSN) is an essential interoceptive pathway that is connected to every level of the body. Its intricate genetic coding provides sustenance for physiological processes, including controlling blood pressure and respiration. Electroacupuncture (EA) is a proven surface stimulation therapy that can regulate vagal nerve activity, which can effectively prevent cardiovascular diseases. A growing number of studies have concentrated on the mapping of VSN codes, but little is known, and the physiological background of how EA influences interoceptive has not been fully explored.

**Method:**

Here, we incorporate the hypothesized interaction among EA targets, VSNs, and the heart. This offers suggestions for using a versatile and focused EA strategy to modify vagal interoceptive awareness to enhance cardiovascular conditions. We first clarified the major role of vagal nerve in the control of cardiac activity. Additionally, we clarified the multidimensional coding pattern in the VSNs, revealing that the targeted control of multimodal interoceptive is the functional basis of the synchronization of cardiovascular system.

**Finding:**

We propose a strategy in which EA of the VSNs is employed to activate the interoceptive loop and reduce the risk of cardiovascular disease.

## Graphical abstract

Mechanisms by which electroacupuncture (EA) drives vagal interoceptive to maintain cardiovascular activity. Vagal interoceptive can be a critical process for sensing, receiving, integrating, and down‐transmitting information, which is crucial for sustaining physiological activities, including blood pressure homeostasis. EA can manage cardiovascular disease in multiple ways by stimulating sensory afferents, facilitating central integration, and modulating visceral efferents. The sensory nerve–muscle junction is postulated to play a pivotal role in mediating the component of EA stimulation that detects noxious information and initiates the subsequent signaling cascades. These signals are then synthesized by central nuclei, such as the nucleus tractus solitarius (NTS), before being relayed to cardiac tissue through the vagus nerve. The precise modulation of multimodal vagal endoreceptors is fundamental to the cardiovascular system's capacity to exhibit varied responses to the reception of EA stimuli, thereby facilitating a nuanced regulatory function in cardiovascular dynamics.

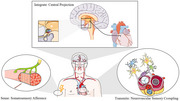

## 1 Introduction

Internal perception is a process of gathering, communicating, and synthesizing information within the body's nerve system, which is impacted by the representations of internal organs, as opposed to exterior senses like smell and sight (Weng et al. 2021). Zhao clarified the three essential distinctive signals that make up the vagal sensory nerve's (VSN) multidimensional patterns, which encode internal organs, tissue layers, and stimulation mode (Zhao et al. 2022). This significant research initially uncovered the heterogeneity of VSN and provided insight into the mechanism of diversity promoting sensory coding at the system level in different dimensions. The VSN is extensively connected to the heart, lungs, pancreas, and other essential organs. VSNs are able to adjust to the morphological and functional characteristics of the various organs, exhibiting a varied molecular structure or anatomical distribution, thus enabling the transmission of specific information (Prescott and Liberles 2022). Vagal nerve stimulation (VNS) is a widely accepted therapeutic approach to heart failure, as it aids in the improvement of cardiovascular tone and the control of sympathetic nerve activity. In addition to altering parasympathetic efferent outflow, vagal nerve transection stimulates the afferent neural fibers in the stimulated trunk (Yamakawa et al. 2015). These findings offer potential strategies for modulating VSN activity and managing internal system responses to external stimuli (such as the ear, which has a wide distribution of vagal sensory endings) (Badran et al. 2018). Investigating multiple parameters and alternative stimulus targets can refine the process of neural regulation.

Electroacupuncture (EA) is an emerging therapy that modulates neural activity, which can protect myocardial ischemia reperfusion injury through optimizing endoplasmic reticulum stress (Wang et al. 2021; Xiao et al. 2020), although the physiological processes are unknown. The majority of existing EA clinical trials in cardiovascular‐related disorders are modest non‐multicenter clinical trials, and no large‐scale EA clinical trials for cardiovascular‐related diseases have been reported, with only a few research studies on effective acupuncture point selection based on biological effects and causes. EA is an innovative approach for manipulating the autonomic nervous system. The vagal nerve can be stimulated through the activation of its afferent fibers (Liu et al. 2021), such as those located in the ear, to facilitate somatosensory autonomic reflexes that can regulate the body's systems. Thus, utilizing EA to activate the VSNs could be a potential solution for cardiovascular diseases. The neuronal circuit reflex activated by EA is specific to the somatic region, and the response to the stimulus can vary depending on the parameters used (Liu et al. 2020). This implies that the distribution of nerve endings and the activated patterns can influence the therapeutic effects of the treatment. Therefore, enhancing the interoceptive coding mode facilitates the selection of the relevant stimulus to precisely activate the vagal interoceptive signal on cardiovascular.

## 2 The Function of Vagal Nerve: A Vital Vehicle for Coordinating the Intrinsic Cardiac Nervous System

Various routes in the brain provide real‐time monitoring of cardiovascular health, and the center is able to integrate multiple linkages to control autonomic outflow and inflow to facilitate response to physiologic perturbations. The brain can be activated by psychogenic stimulus, and the intrinsic cardiac nervous system (ICNS) can also be susceptible to inhibitory inputs from the brain due to the bidirectional interaction between them (Aksu et al. 2021). The structural foundation for communicating the integration of information between the two is provided by the vagal nerve, although there is insufficient elucidation of its function in ICNS.

### 2.1 Innervation of the Vagal Nerve in the Heart

**FIGURE 1:**
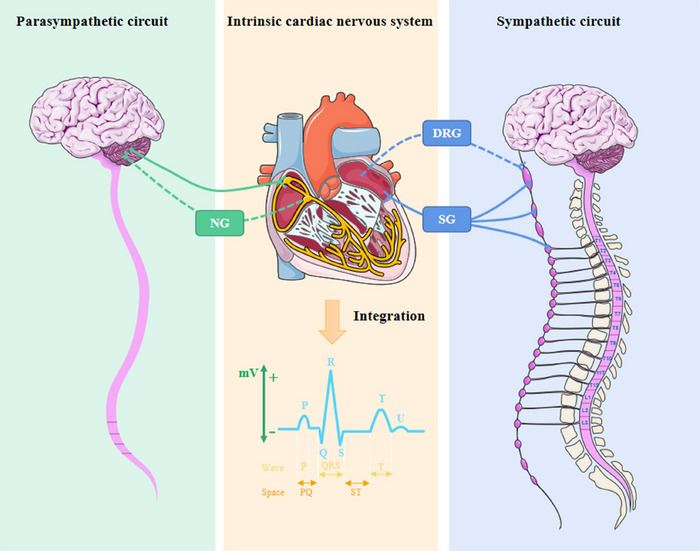
Innervation loops of the heart. Different nerve fibers can coordinate signals to maintain cardiovascular physiological activity. The dotted line represents the afferent nerve, and the realization indicates the efferent nerve. DRG, dorsal root ganglion; NG, nodal ganglion; SG, stellate ganglion.

The nervous system that innervates the heart is complex, in which ICNSs are signal relay stations with rapid integration ability and communicate information with peripheral autonomic nerves (Wake and Brack 2016). The neurophysiology of cardiovascular disorders is based on aberrant ICNS alterations, which are located in epicardial adipose tissues and are connected to sympathetic paraspinal, dorsal root, and nodose ganglion via synaptic connections, resulting in the formation of both long and short feedback loops (Figure 1) (Goldberger et al. 2019). Although the sympathetic nerve is the principal transmitter of sensory afferent fibers from the heart and pericardium, numerous fibers can also be transferred by the vagal and phrenic nerves. For instance, the ICNS is in charge of interpreting parasympathetic physiological information, primarily from the cervical pre‐ganglionic vagal nerve fibers, and organizing systemic reactions throughout the heart (Yamakawa et al. 2015). Under pathological circumstances such as pre‐myocardial ischemia, ICNS and peripheral autonomic nerves can engage in adaptive cross‐talk to prevent pressure overload from exacerbating the illness (Hausenloy et al. 2019). The sympathetic nerve is the main nerve involved in the innervation of the heart, yet the vagal nerve should not be disregarded, particularly its capability to recognize pressure in the carotid sinus and the sinoatrial node (Gierthmuehlen, Plachta, and Zentner 2020). As opposed to sympathetic neurons, parasympathetic neurons are more concentrated in particular areas of the heart, especially the sinoatrial node and atrioventricular node (Christensen et al. 2022). The parasympathetic nerve cells possess a much simpler messenger system than the sympathetic nerve, which is evident in the speed and immediacy of the heart's response to the stimulation of the parasympathetic nerve branch, as well as the transmission of the termination signal (Sloan and Cole 2021). The ICNS cells are capable of receiving both low‐ and high‐intensity stimulations from the cervical vagal efferent fibers and the heart (Ottaviani et al. 2020). This intricate connection between the cardiac nervous system and the stimulation response is essential for the proper functioning of the nervous system. The heart is able to sustain its optimal performance throughout the entire level under physiological conditions, with the different tissues making the necessary adjustments. The intricate and layered nervous system underscores the dynamic nature of its regulation of the heart. Angiotensin (Ang) II receptors, for instance, can modulate vagal activity by acting at the level of nerve endings and are found throughout the parasympathetic nerve (Shanks and Ramchandra 2021). The positive benefits of angiotensin receptor blocks like hypertension may be partially mediated through effects on parasympathetic neurons due to the tonic inhibitory effect of endogenous Ang II on cardiac vagal tone (Shanks and Ramchandra 2021). The vagal end organ terminals may also be trafficked and are found in the nodose ganglia cell bodies (Allen et al. 1988). These investigations imply that one of the biological foundations for vagal communication between the brain and the ICNS is the bidirectional translocation of Ang II binding site receptors along the vagal nerve. The composite neural network collaborates to feedback information, thereby creating the spatial and temporal characteristics of cardiac activities. The tetratricopeptide repeat‐containing Rab8b (TRIP8b) vagal deletion, for instance, affects the conduction properties of atrial cardiomyocytes and atrioventricular node conduction but not ventricular activity (Scherschel et al. 2021); hence, the predominance of sympathetic/parasympathetic innervation in a particular area is a critical factor in deciding the treatment plan. When physiological equilibrium is disturbed, the dynamics of each system and their interaction have yet to be elucidated.

### 2.2 VSN Genes Encode for the Regulation of Partial Cardiovascular Function Characterization

Excessive activity of the sympathetic nervous system and a reduction in the activity of the vagal nerve are considered the key pathological factors that cause ventricular remodeling and heart failure. This deterioration of heart failure will then further exacerbate the sympathovagal imbalance, forming a vicious cycle (Sant'Anna et al. 2021). Therefore, targeting the autonomic nerves, particularly the vagal nerve, is a vital component of the treatment and prevention of heart failure and other cardiac diseases. For example, the optogenetic stimulation of vagal preganglion neurons was demonstrated to be effective in averting a decline in ejection fraction due to myocardial infarction (Machhada et al. 2020). The sympathetic nervous system provides close support to vascular activity, which is further modulated by the vagal nerve. This allows for the release of adenosine triphosphate from sensorimotor nerve endings, which in turn elicits antidromic reflex activity and relaxes the vascular system (Burnstock 2017). The vagal nerve is equipped with the ability to sense changes in oxygen levels in the blood, observe the flow and chemical makeup of the blood, provide timely and accurate feedback, and maintain alterations in blood pressure and even the rate of respiration (W. G. Chen, Schloesser, et al. 2021). Applying carefully directed electrical impulses to the neck area can access the potential of the vagal sensory and motor nerve fibers, creating a beneficial effect on the failing heart (Bucksot et al. 2020). The intimate connection between VSNs and blood vessels implies that the brain may utilize multiple pathways to regulate cardiovascular activity. The VSNs that are indicated by glucagon‐like peptide‐1 receptor (*glp‐1r*) and G protein–coupled receptor 65 (*gpr65*) are distinguishable from the VSN fibers arising from the aorta and leading to the brain as they do not possess a distinctive characteristic of tightly encircling the arterial walls. It appears that genetic coding information can be used to differentiate between functional representations of VSNs. Although different types of vagal sensory terminals have been identified, including “Ruffini type,” “end‐net,” and “flower spray” terminals. This gene‐profiling strategy provides a more in‐depth understanding of intrinsic neuron characteristics than traditional studies that focus on the difference of terminal position or orientation. The vagal nerve has a broad reach, with its nerve endings most densely populated in the flower spray terminal, extending along the aortic arch (Min et al. 2019). Data from Mc4r‐p2a‐Cre mice indicate a slight reduction in blood pressure following Mc4r photogenetic stimulation, which is in line with the effects of aortic inhibition. However, no major alteration in heart rate was observed, which is distinct from the outcome of baroreceptor stimulation (Min et al. 2019). The baroreceptor, however, is primarily one of the markers by phox2b, or Piezo1/2 (Zeng et al. 2018). Transient receptor potential cation channel (Trpv1)‐positive VSNs were predominantly found in the outer layers of the heart, whereas Sst and Gpr65‐positive VSN endings were located in the deeper layers (Zhao et al. 2022). The functional map of the VSNs transmitting stimulus signals to different tissue levels in the heart is limited by the relative position of the surface‐luminal axis of different gene‐coding lineages of this VSN terminal, which follows a gradient of morphogenesis. Clarity on the intrinsic qualities of the VSN will not only facilitate the understanding of the dominant target organs’ characteristics and functions but also enable the development of targeted stimulation approaches. This article delves into the role of the VSN in the development of cardiovascular pathology, elucidating the importance of maintaining a balance between the sympathetic and vagal nerves. Additionally, it provides insights into the development of clinical decision‐making tools and potential treatment strategies. The efficacy of non‐targeted vagal nerve block has been established, yet our understanding of the involvement of sensory and motor neurons is incomplete. This may impede the exploration and implementation of this technique in clinical practice. The VSN has a major influence on cardiovascular regulation, and furthering our knowledge of its composition will help us to identify more effective ways to control and vagal interoceptive.

## 3 Novel Strategies for the Management of Cardiovascular: Modulation of Vagal Interoceptive

The neurons of VSNs can adjust to changes in physiological responses, such as changes in cardiac input volume, in real time by enhancing discharge patterns. VSNs can synergistically influence a range of cardiovascular reflexes. In order to comprehend the role of VSNs in cardiovascular activity, it is essential to describe the physiological responses and sensory patterns of vagal interoceptive.

### 3.1 Strategic Positioning of Vagal Interoceptive in the Cardiovascular Center

The diversity of the coding system of the VSN endings at different organizational levels is the molecular foundation for its multiple responses and synchronized response threshold/sensitivity. Interoception is primarily assessed through the cardiovascular system, with an emphasis on accuracy (Garfinkel et al. 2015). Transcutaneous VNS has been found to be capable of modulating endosensory processes, thereby improving the accuracy of cardiac interoceptive perception (Villani, Tsakiris, and Azevedo 2019). This implies its potential to be utilized as a tool for studying the connection between the body and the brain. Along with the nervous system, the vasculature is one of the processing systems engaged in interoceptive processes. The arterial region innervated by pressure‐sensing neurons is highly elastic, allowing it to be sensitive to the fluctuations of blood flow pulsation and to pick up on even the slightest changes in pulse signals (Guyenet 2006). The second‐order neurons in the caudal part of the nucleus tractus solitarius (NTS) can be stimulated by sensory input from the visceral vagal nerve and by instructions from higher brain areas (Holt 2022). The NTS architecture of multiple dimensions facilitates the modulation of cardiovascular pathways in a unified manner. The malleability of baroreceptors allows for the vagal sensory transmission of information to the NTS, a pivotal junction of signal modulation that combines impulses and regulates heart rate and blood pressure (Ho et al. 2020). The dorsal horn of the spinal cord sends additional sensory information to the NTS, possibly including signals for painful and tactile stimuli. This is due to the cardiac vagal pre‐ganglionic and pre‐sympathetic neuronal circuitries anatomical placement next to the ventral–lateral area of the medulla oblongata (Holt et al. 2019). Cardiac and pulmonary signals can be combined and sent to the dorsolateral solitum nucleus through the VSN pathway (Cutsforth‐Gregory and Benarroch 2017), demonstrating the intricate structure of the lateral vagal interoceptive. The modulation of respiratory sinus arrhythmias is carried out by cardiac vagal pre‐ganglionic neurons, which are found in the long lateral medulla. Their efferent pattern encodes rhythmic respiration‐related alterations (Spyer 1994). The synaptic coupling formed by these neuronal circuits is the structural basis for the heart to rapidly sense respiratory changes, such as receiving feedforward signals from respiratory activity to modify cardiac output. This means that external stimulus signals or internal sensory coding can activate both organs simultaneously, leading to communication cross‐talk. Engaging in meditation or simply practicing deep and rhythmic breathing can have a calming influence on the heart by adjusting the depth of the breath (Paccione et al. 2022). Meditation can lead to a restraint in overall vagal modulation and an enhancement in sympathetic modulation and baroreflex activity (Brown et al. 2021). A gene‐based analysis of the anatomy showed that Npy2r and P2ry1 neurons are widespread in the airways and partially connecting to the heart (Chang et al. 2015). Alongside other varieties of VSNs, they transmit signals to the ganglion, allowing the VSN to coordinate cardiopulmonary activities. It appears that the physiological properties of VSNs are not only reliant on their molecular structure but also influenced by their strategic placement in the body. For instance, the spherical cells in carotid sinus simultaneously assess ventilation, oxygen exchange, and blood flow status, transmitted comprehensive information to the medulla oblongata and NTS, and coordinated cardiovascular vagal activities.

### 3.2 Multidimensional Feedback Patterns of Vagal Interoceptive in the Heart

**FIGURE 2:**
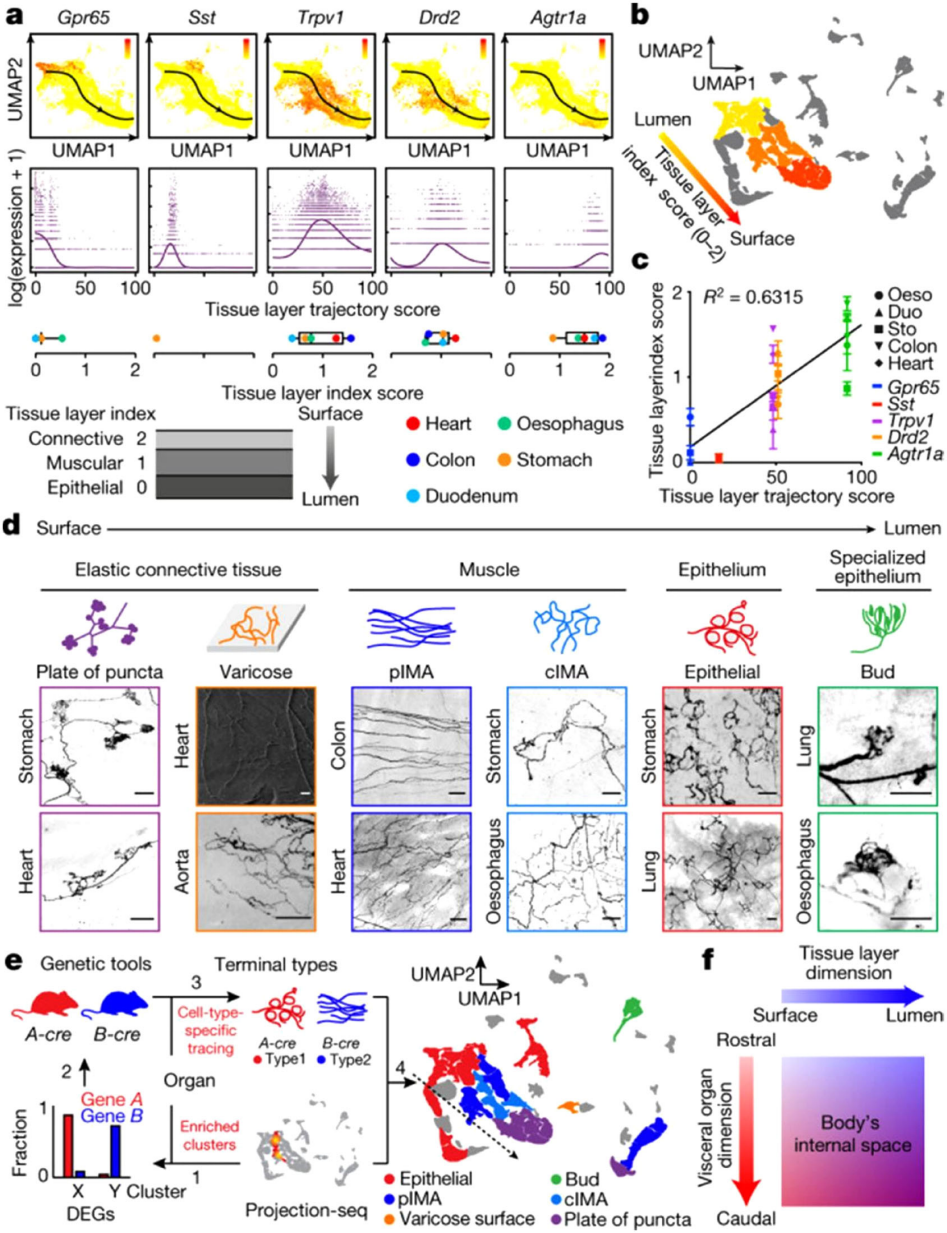
A “tissue layer” dimension coding VSN ending locations and structures. (a) UMAP plots of identified DEGs (top) and their expression measures (middle) along a “tissue layer” trajectory. Bottom, DEG+ VSN ending locations, quantified as “tissue layer” index score in corresponding DEGtdT mice (mean, number of mice: Gpr65‐oesophagus, 3; Gpr65‐stomach, 7; Gpr65‐duodenum, 4; Sst‐stomach, 5; Trpv1‐oesophagus, 3; Trpv1‐stomach, 7; Trpv1‐duodenum, 4; Trpv1‐colon, 4; Trpv1‐heart, 10; Drd2‐oesophagus, 3; Drd2‐stomach, 6; Drd2‐duodenum, 3; Drd2‐colon, 2; Drd2‐heart, 6; Agtr1a‐oesophagus, 4; Agtr1a‐stomach, 12; Agtr1a‐duodenum, 4; Agtr1a‐colon, 3; Agtr1a‐heart, 6). (b) UMAP plot of VSN clusters, colored by average tissue index determined in Gpr65tdT (F1–F4 clusters; golden), SsttdT (F5 cluster; yellow), Drd2tdT (J2–J4, H2, H4, and I1 clusters; orange), and Agtr1atdT (I2 and I4–6 clusters; orange–red) mice, showing a continuous trajectory coding tissue layers along the organ's surface–lumen axis. (c) Correlation between mean “tissue layer” trajectory score of DEG+ VSNs and their “tissue layer” index score in corresponding DEGtdT mice (mean ± s.e.m.; n as in (a)). Linear regression R2 Linear r. (d) VSN ending types characterized in Vglut2tdT mice show stereotypical structures along various tissue layers across multiple visceral organs. Scale bars, 100 µm. (e) Projection‐seq‐guided anterograde tracing (schematic illustration, left) reveals genetic identities of stereotypical VSN ending types illustrated on the UMAP plot (right). VSN clusters forming various VSN ending types followed the “tissue layer” trajectory well (dashed arrow). (f) Model for combinatorial coding of the body's internal space in VSNs using a 2D genetic matrix. *Source*: Figure adapted from Zhao et al. (2022) with permission from Nature.

The vagal nerve is equipped with an intricate and intricate feedback cycle. Most of the afferent fibers of the vagal nerve are second‐order neurons, responsible for detecting signals sent by upstream cells, such as taste cells and intestinal secretory cells (Moreno‐Dominguez et al. 2020). Additionally, some VSNs are first‐order neurons that directly sense stimuli from internal organs (Han et al. 2018). Glomus cells, being the first‐order chemosensory cells of the carotid body, are capable of sensing multiple stimuli (Ortega‐Saenz and Lopez‐Barneo 2020). They form chemical and electrical synaptic connections with dopaminergic and acetylcholinergic nerves, among others, and also bridge communication between glossopharyngeal nerves and the brain/body regarding changes in blood flow (Torrealba and Alcayaga 1986). The anatomical position of neurovascular coupling and the multilevel feedback function form a structural basis for the nervous system to observe the circulation of major arteries and regulate the supply of blood to vital organs. The vagal nerve endings are distinct in the data they encode and can be identified through their genetic markers, which are associated with different anatomical connections and physiological functions and send remote projections to different brain stem targets. Drd2^+^ VSNs were expressed in the myocardium of the heart and were present in abundance in all gastrointestinal organs. Agtr1a^+^ VSNs, in contrast, were mainly located in the outer myometrium or epicardial layer (Zhao et al. 2022), implying that the initial projection trajectory of the vagal sensory system architecture may follow a general rule rather than demonstrating the particular projection between organs at the outset. Even with such complex coding patterns, all cardiopulmonary inputs can be integrated and projected to the dorsolateral solitum nucleus by targeting the VSN pathway, and this property also indicates the multidimensional construction of vagal interoceptive laterally. These offer the potential to manipulate vagal interoceptive. The gene expression profile showed the diversity of VSNs (Figure 2), which strongly suggested that VSNs were not simply “on” or “off” but could independently coordinate the physiological functions of discrete organs (Bai et al. 2019). The coding representation of vagal interoceptive provides a directional strategy for selective manipulation of neurons to modulate cardiovascular function.

## 4 Potential Mechanisms of EA in Managing Cardiovascular Disease: Utilizing Adaptive Stimulation of the Vagal Nerve

**FIGURE 3:**
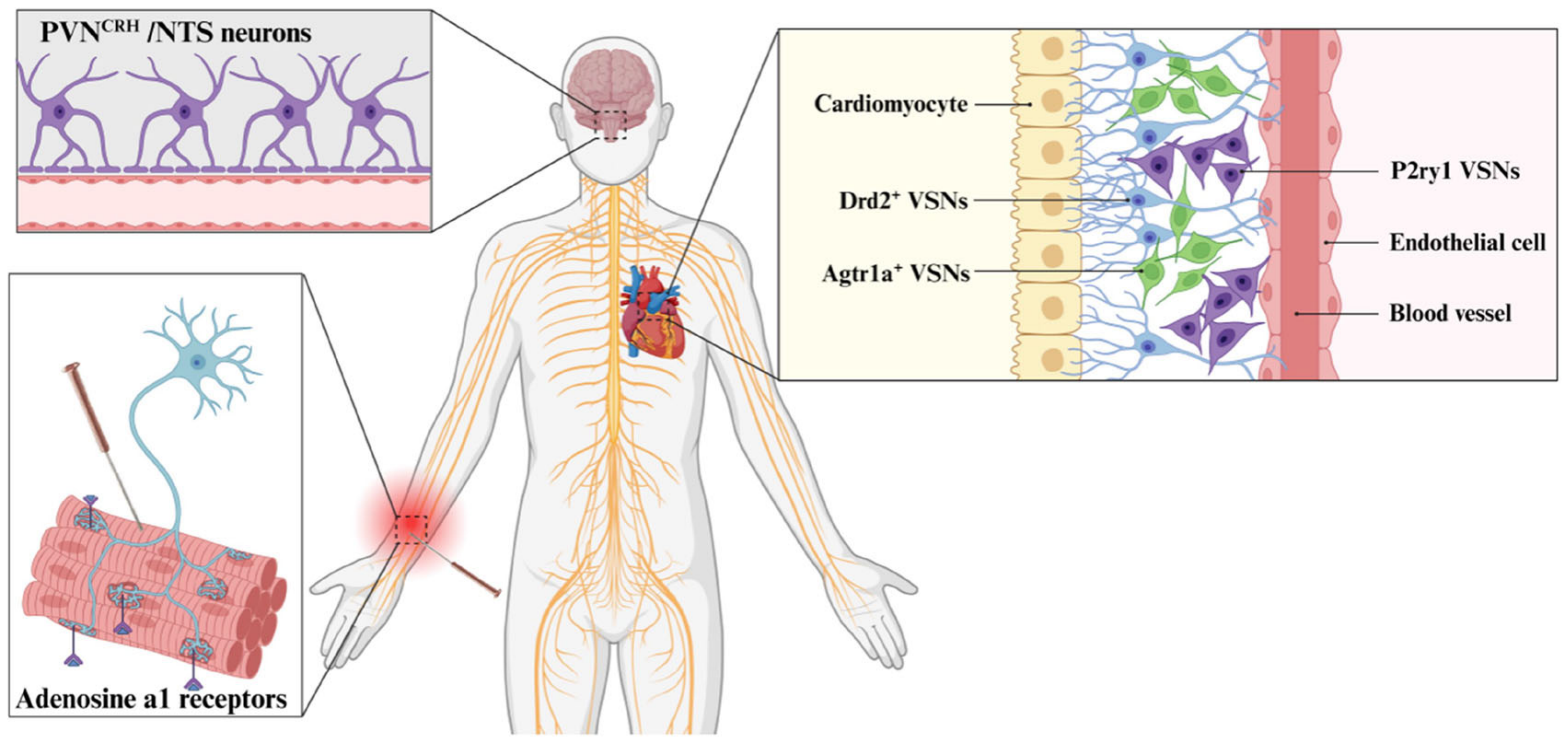
Potential vagal endoreceptor neural basis for EA regulation of cardiac function. EA at Neiguan acupoint (PC6) activates adenosine a1 receptors on somatic afferent nerve endings, generating nerve impulses and triggering the EA effect. Sensory signals are transmitted to the central NTS and PVN^CRH^ in nuclei that regulate vagal output. Ultimately, different anatomical levels of the cardiovascular system are targeted by Drd2^+^, Agtr1a^+^, Npy2r^+^, and P2ry1^+^ VSNs to produce different EA effects. CRH, corticotropin‐releasing hormone; EA, electroacupuncture; NTS, nucleus tractus solitarius; PVN, paraventricular nucleus; VSNs, vagal sensory nerves.

By stimulating local sensory nerves and other afferents in the acupoint, EA can set off a cascade of reactions in the organism across multiple systems and tissues. This shows that EA has the capacity to alter VSNs’ function patterns, indirectly raising the prospect that EA can treat cardiovascular diseases by modifying vagal interoceptive (Figure 3).

### 4.1 EA Activation of Somatic Sensory Afferent Nerve: Vital Functional Input Integration of the Heart

Accumulating evidence suggests that transcutaneous auricular VNS (taVNS) influences activity in solitary and parabrachial nuclei, the primary brainstem relays for the transmission of visceral sensory afferents to the insula (Poppa et al. 2022). Thus, examining the effect of external stimuli on the integration of internal perception will be a worthwhile contribution to the clinical treatment program. When the skin is scratched or cut severely, satellite cells associated with the dorsal root ganglion may begin to develop. These cells are responsible for sustaining sensory neurons with necessary nutrients and energy (Petrella et al. 2020). The somatic sensory afferent nerve fibers enable the transmission of mechanical stimulation or injurious signals from the skin to the center, where they are integrated and trigger reactions such as tissue repair and inflammation control (Christie et al. 2015). This offers a novel outlook for the vagal interoceptive receptors to comprehend mechanical stimulus signals, decode them, and direct subsequent systemic reactions, as well as initiate surface stimulation. These raise the possibility of treatment approaches that start with the skin to treat cardiovascular disorders. On the basis of this neurological basis, the benign impact of auricular acupuncture on the heart mimics the auriculo‐cardiac reflex (Lin et al. 2011). EA has the ability to affect the nervous system by stimulating certain areas on the surface of the body, such as activating adenosine a1 receptors on nerve endings, generating nerve impulses, and triggering acupuncture effects (Wang et al. 2020). EA has been proven to be effective in reducing long‐term cardiac sympathetic stress, enhancing vagal tone, and providing protection for the heart (Xiao et al. 2020). Research conducted at the central level has revealed that the vagal nerve can alter the activity of neurons associated with the paraventricular nucleus (PVN), which possess typical ganglion innervation properties (Tjen‐A‐Looi et al. 2022). This indicated that the local surface of the body can be connected to target organs through specific neural pathways, allowing the autonomic nervous system to re‐establish equilibrium via the superior spinal cord center, thus optimizing the body's state and improving the progression of the disease. Following the deep axis of the body, the sensory neurons of the vagal nerve communicate with the body surface and the viscera, forming a wide‐ranging and powerful body–brain connection, which is one of the physical bases of surface medicine such as EA. EA at Neiguan acupoint (PC6) has the ability to ameliorate bradycardia, control vagal‐induced NTS activity through a γ‐aminobutyric acid–ergic pathway, and control the response of the cardiovascular system (Tjen‐A‐Looi, Guo, and Longhurst 2014). EA pretreatment could inhibit PVN^CRH^ neurons, which offers fresh perspectives on the application of acupuncture in the management of cardiovascular disease (Zhou et al. 2024; Zhu et al. 2023). This also implies that stimulation of certain areas of the body can enhance the performance of the target organ. Head's zones also law outlines the common driving force for nerve integration between body surface and heart. Cardiac afferent neurons are capable of detecting mechanical, chemical, or multiple stimuli and can transmit peptides such as substance P and calcitonin gene–related peptides (Shivkumar et al. 2016). This can cause an increase in local skin release after EA (Xu et al. 2022), which is then transmitted through the VSN and is thought to play a role in the remodeling of the ventricles (Wang et al. 2014). By combining multiple systems and multiple targets, EA is able to act as a regulator of both the nervous and circulatory systems. The stimulation signal of EA can either activate or inhibit certain nerve structures, thus regulating the system and producing a local effect on the body surface and a targeted effect on the organ. Research studies into the neurophysiological properties of EA have shed light on its potential use in the treatment of cardiovascular diseases, including heart failure.

### 4.2 EA Modifies Neurovascular Sensory Interaction: Alternate Route for Cardiac Input Regulation

Vagal intrasthesia utilizes massively parallel encoding of location information and stimulation mode (Zhao et al. 2022), which allows for external stimulus information, such as hemodynamic or peristaltic signals, to be translated into encoded information that is processed by the internal nervous system. This provides a foundation for surface medicine therapy. EA is capable of not only normalizing autonomic tension but also preserving the autonomic nerve output linked to hemodynamic stability (Li et al. 1998). In other words, EA does not modify the hemodynamic baseline. Studies have demonstrated the presence of sensory neurites related to the ICNS at the atrioventricular junction and on the outer membrane of major vessels (e.g., coronary arteries) (Lizot et al. 2022), supporting that EA may have a protective effect on the heart by regulating both the nervous and circulatory systems. EA may enhance aberrant nerve activation close to ischemia foci and develop into one of the prospective therapy options for neurovascular illness (Li et al. 2022). Piezo1 and Piezo2 are parts of the molecular mechanisms behind the vagal interoceptive, which senses blood pressure and acts as pressure sensors for the heart's mechanical work (Jiang et al. 2021). EA has been demonstrated to be an effective method for improving hemodynamics, modulating blood flow in areas of ischemia, altering coronary microvascular tension and repairing nerves, and providing potential for vagal interoceptive regulation (Silva et al. 2020; Zhang et al. 2021). Meanwhile, the coding gene template in different tissue layers and organs is heterogeneous, which makes VNS considered a multidimensional functional unit. This could explain why uniform stimulation of distinct parts of the body surface yields distinct outcomes. Using topological mapper methods, Feng found that sympathetic neurovascular networks were denser in the acupoint compared with surrounding non‐acupoints (Hu et al. 2021). If the benefits of acupoint neurovascular coupling are further explained, the basis for the local and systemic biological effects of EA in the treatment of cardiovascular disease can be understood better. Furthermore, VSNs may exhibit this variability. Wang also demonstrated that EA at PC6 was effective in alleviating myocardial ischemia‐reperfusion; however, the same effect could not be replicated when EA was applied to other body parts (Wang et al. 2021). In terms of the quantitative‐effect relationship, the difference in acupuncture depth impacts the therapeutic effect due to variances in the distribution of nerves and various types of receptor cells at varying depths of the local tissue at the acupoint (L. Chen, Wang, et al. 2021). Different stimulation modalities may cause varied signal transduction and expression for the coding features of EA. This raises the possibility of surface medicine being a suitable remedy for cardiovascular ailments, although its success is likely to be location‐specific due to the differences in the way VSN messages are transmitted and reintegrated (Garfinkel et al. 2016). Ultimately, EA has the potential to be beneficial by controlling the activity of the sensory nervous system, both in terms of afferent and efferent pathways. A comprehensive understanding of this process will be beneficial in ensuring the safety and effectiveness of its clinical applications.

## 5 Conclusion and Future Perspectives

Categorization of VSNs is traditionally done by analyzing their physical characteristics, developmental background, and sensitivity to external stimuli. The utilization of gene sequencing has enabled researchers to further investigate the variety of characteristics of sensory neurons. The gene‐coding spectrum of this particular sensory attribute and its anatomical position are mutually dependent, allowing for an investigation into the precise stimulation and regulation of target organs and the utilization of interorgan communication. Meditation can have a regulating effect on the cardiopulmonary system. The physiological basis for this is the vagal interoceptive loop regulation; for instance, photogenetic stimulation of P2ry1 neurons can significantly reduce respiration without affecting heart rate, which is indicative of the precise interoceptive encoding pattern of the vagal nerve. The perspective from local to global region provides multimodal interoception, indicating the strategic positioning of the VSN to the visceral projection pathway. This provides the neurological basis for the precision of cardiovascular regulation. The vagal sensory afferent nerve facilitates the transition of internal signals in accordance with the cutaneous information received from external stimuli. Evidence has indicated that taVNS and EA are viable approaches for managing cardiovascular health. EA acts on the nervous system; the multimodal VSN is the direction of the research of the molecular mechanism strategy of EA regulation of the cardiovascular system in order to optimize the shortcomings of existing therapies. Investigating the physiological basis of interoception in relation to the cardiovascular system, understanding the transmission and transduction of sensory signals to internal organs, and exploring the potential of EA to stimulate the encoded signals of neural pathways will provide a basis for the study of EA in the prevention and treatment of cardiovascular diseases. Further research studies into the genetic coding of VSNs could lead to the augmented comprehension of individual afferent or efferent neural pathways and more effective therapeutic approaches for treating illnesses.

## Author Contributions

**Yun Liu** and **Tiancheng Xu**: conceptualization. **Yun Liu** and **Tiancheng Xu**: Writing–original draft preparation. **Bin Xu** and **Zhi Yu**: Writing–review and editing. All authors read and approved the final manuscript.

## Conflicts of Interest

The authors declare no conflicts of interest.

### Peer Review

The peer review history for this article is available at https://publons.com/publon/10.1002/brb3.70076.

## Data Availability

Data sharing is not applicable to this article as no datasets were generated or analyzed during the current study.
